# Virulence and Drug-Resistance of *Staphylococcus aureus* Strains Isolated from Venous Ulcers in Polish Patients

**DOI:** 10.3390/ijerph18094662

**Published:** 2021-04-27

**Authors:** Mateusz Gajda, Emilia Załugowicz, Monika Pomorska-Wesołowska, Tomasz Bochenek, Barbara Gryglewska, Dorota Romaniszyn, Agnieszka Chmielarczyk, Jadwiga Wójkowska-Mach

**Affiliations:** 1 Department of Microbiology, Faculty of Medicine, Jagiellonian University Medical College, Jagiellonian University, 31-121 Krakow, Poland; d.romaniszyn@uj.edu.pl (D.R.); agnieszka.chmielarczyk@uj.edu.pl (A.C.); jadwiga.wojkowska-mach@uj.edu.pl (J.W.-M.); 2 Doctoral School of Medical Sciences and Health Sciences, Jagiellonian University Medical College, Jagiellonian University, 31-530 Krakow, Poland; 3 Faculty of Biology, Jagiellonian University, 30-387 Krakow, Poland; emilia.zalugowicz@gmail.com; 4 Department of Microbiology, Analytical and Microbiological Laboratory of Ruda Śląska KORLAB NZOZ, 41-703 Ruda Śląska, Poland; monikapw@op.pl; 5 Department of Nutrition and Drug Research, Faculty of Health Sciences, Jagiellonian University Medical College, Jagiellonian University, 31-066 Krakow, Poland; mxbochen@cyf-kr.edu.pl; 6 Department of Internal Medicine and Gerontology, Faculty of Medicine, Jagiellonian University Medical College, Jagiellonian University, 31-531 Krakow, Poland; bgrygle@su.krakow.pl

**Keywords:** drug resistance, leg ulcers, *Staphylococcus aureus*, virulence

## Abstract

Infected chronic venous ulcers (VUs) represent a major health problem. We analysed the aerobic microbiome in the VUs, the virulence, and drug-resistance of *Staphylococcus aureus* (SA) strains. Swabs from 143 outpatients and inpatients Polish subjects were collected. SA strains were tested for drug sensitivity using a phenotyping method and for methicillin-resistant SA (MRSA) and macrolide-lincosamide-streptogramin B (MLSB) resistance using PCR. We analysed virulence genes, the genetic similarity of strains, and performed Staphylococcal cassette chromosome *mec* typing and Staphylococcal protein A typing. SA was isolated as a single one in 34.9% of cases, 31.5% paired with another pathogen, and 33.6% *S. aureus* combined with at least two other strains. The majority of SA isolates (68.5%) possessed the virulence lukE gene. Drug resistance was significantly common in hospitalised than in ambulatory patients (OR 3.8; 95%CI 1.8–7.91). MLSB (altogether in 19.6% isolates) were observed mostly in non-hospitalised patients (OR 9.1; 95%CI 1.17–71.02), while MRSA was detected in 11.9% of strains equally. Hospitalisation and patient’s age group (aged > 78.0 or < 54.5 years) were significant predictors of the multi-drug resistant SA (MDR-SA). Over 30% of the infected VUs were associated with multi-species biofilms and presence of potentially highly pathogenic microorganisms. Elderly hospitalised patients with chronic venous ulcers are prone to be infected with a MDR-SA.

## 1. Introduction

Venous ulceration (VU) of the lower extremity is an open sore in the lower leg’s skin, resulting from chronic venous insufficiency and high blood pressure in the leg veins [1]. The persistent venous hypertension causes retention of a high-protein fluid within tissues, which triggers an inflammatory process and activates the leucocytes. The resulting destruction of skin and subcutaneous tissue is observed as an ulceration. Leg ulcers have been most clearly defined by the WHO [2,3,4]. The prevalence of active leg ulcers is between 1.5 and 3.0 per 1000 population, but it rises with age to reach about 20 in people aged more than 80, as estimated on the British population. [5] Similar estimates have been made for the American adult population [6,7]. In a Polish study, involving more than 40,000 patients of general practitioners, venous ulcers in general were reported in 1.52% of patients, and the active ones—in 0.55% [8]. The latest Polish angiological recommendations indicate the prevalence of venous ulcers at the level of about 3.0 per 1000 [9].

Chronic leg ulcers may also be caused by other pathologies, e.g., arterial insufficiency, mixed arteriovenous disease, diabetes and rheumatoid arthritis, or, less frequently, autoimmune diseases, cancer, or tropical diseases. However, 70–81% of them are connected with venous insufficiency [10,11]. Due to a relatively common prevalence, chronic and painful character, as well as a long-term and complex treatment, venous leg ulcers constitute not only a challenging therapeutic problem, but also a socio-economic burden. With the aging population and the rising proportion of patients with other co-morbidities (diabetes mellitus, atherosclerosis) in the population, venous leg ulcers and their complications are going to pose a significant problem for healthcare systems.

The venous insufficiency usually lasts for years, and therefore recurrences can be observed in spite of a successful treatment. The treatment is based mostly on compressiotherapy, vein surgery, and skin grafting, but also on the local and systemic antibiotic therapy [3]. A common etiological factor of chronic leg ulcer infections is *Staphylococcus aureus*. It belongs to the natural microflora of the human mucosa, but at the same time it has many virulence features, which may contribute to pathogenicity and local infections. The data shows that approximately 30% of the human population is colonized with *S. aureus* [12]. The authors indicate the presence of this pathogen in wounds from 20% to over 50% of cases [13,14]. It evades host immune defences and facilitates the translocation to the bloodstream. Staphylococcal virulence factors are crucial to the invasive character of the strains. Panton-Valentine leucocidin, E and D leucocidin, and α-haemolysin have the capacity to lyse host cells. Exfoliative toxins facilitate a bacterial skin invasion, whereas enterotoxins and TSST-1, as superantigens, activate the lymphocyte T-cells. Testing the virulence profile of bacteria seems to be a reliable method of predicting the behaviour of *S. aureus* in wounds [15,16]. *S. aureus* infections of VUs may also develop numerous mechanisms of resistance to antibacterial drugs. According to our recent study, the antimicrobial resistance (AMR) remains a serious problem for the public health in Poland, where the system of monitoring the AMR and appropriate strategies to address the problem remain underdeveloped, while the role of microbiological diagnostics and the efforts to prevent infections are underestimated by physicians.

Polish population is characterized by a high consumption of antibiotics, the highest one of the studied European countries. The total consumption of antibacterials for systemic use, as well as the relative consumption of beta-lactamase-sensitive penicillins, were constantly rising in 2007–2016 [17]. In 2013, the prevalence of Methicillin-Resistant *Staphylococcus aureus* (MRSA) in Poland amounted to 16%, whereas in many European Union (EU) countries it exceeded 25% [18]. The high consumption of trimethoprim and sulfamethoxazole in Poland, accompanied by high levels of antibiotic resistance with MRSA prevalence 17.3%, was described by Pomorska-Wesołowska et al. in 2017 [19].

Considering the increasing antimicrobial resistance and chronic wound prevalence, there is an urgent need to understand the epidemiology and risk factors of *S. aureus* infections. The aim of our study was, then, to analyse the aerobic microbiome and investigate the virulence and drug-resistance of the *S. aureus* strains being an aetiological factor of chronic wound infections of outpatient and inpatient subjects. We also aimed to determine the scale of the problem of venous ulcer infections with *S. aureus* strains, either the multidrug-resistant ones or those carrying genes of extraordinary virulence.

## 2. Materials and Methods

This was a laboratory-based study. The samples were collected between 1 January and 31 December 2013, in collaboration with the Department of Microbiology Jagiellonian University Collegium Medicum, and 2 collaborative microbiological laboratories from the Silesia region: KORLAB NZOZ in Ruda Śląska and St. Barbara Specialised Regional Hospital No. 5, Sosnowiec. All patients with chronic wounds referred to the clinic or wound care unit (e.g., surgery clinic) who agreed to participate in the study were included in the study. Patients with pressure ulcers and under 18 years old were excluded from the study. We have also collected the relevant patient information, i.e., age, sex, place of treatment (ambulatory care or hospitals & long-term care facilities, LTCF) and type of hospitalisation (surgical or non-surgical wards, with LTCF classified as non-surgical wards).

Independent samples were collected from the hospitalised (95 cases) and non-hospitalised (outpatient care, infection treatment in home care, 48 cases) patients after the attending physician had diagnosed an ulcer infection. Chronic wounds were defined as those with a duration of >6 weeks. The infections were classified as “skin infection: cellulitis/soft tissue/wound infection,” in accordance with European Centre for Disease Prevention and Control (ECDC) definitions for long-term care facilities [18].

Susceptibility testing, DNA isolation, polymerase chain reaction (PCR) screening for resistance genes and for virulence factor genes screening, pulsed-field gel electrophoresis (PFGE), *Spa* and SCC*mec* typing were carried out using previously published methods (supplementary material).

### 2.1. Bacterial Isolates

The bacterial strains were collected and identified at the collaborating laboratories. In each case, two swabs were collected: one was used for the direct microscopic examination and the second was put into Amies or Stuart transport medium. Both swabs were, then, taken to the microbiological laboratory. The growth of bacteria was evaluated semi-quantitatively. Isolates were identified using the automated identification system (VITEK 2 COMPACT, bioMerieux, Marcy l’Etoile, France).

Altogether, 143 independent (a single strain was derived only from the first sample collected in the case of the first episode of infection) *S. aureus* strains were isolated. The samples were stored in the Department of Microbiology at the Jagiellonian University Medical College at −70 °C.

### 2.2. DNA Isolation

DNA was extracted from the isolates using the Genomic Mini kit (A&A Biotechnology, Gdynia, Poland), according to the manufacturer’s instructions.

### 2.3. Polymerase Chain Reaction (PCR) Screening for Resistance Genes

MRSA phenotype was determined by the detection of the *mecA* gene in PCR amplification using previously published primers [20]. Genes involved in erythromycin resistance (*ermA, ermB, ermC* and *msr*) were detected by multiplex PCR and amplification of a 456 bp fragment of the *mupA* gene performed by single PCR [21,22]. Relevant positive *S. aureus* ATCC 33591, ATCC BAA-1708 were included. Water was used for the negative control.

The *spa* gene amplicons were analysed by electrophoresis on a 1.5% agarose gel. The sequencing of PCR products was subcontracted to an external laboratory (Genomed, Warsaw, Poland). The nucleotide sequences were analysed to assign the isolates to various types using the *spa* typing website Ridom SpaServer (http://www.spaserver.ridom.de, accessed on 21 February 2014), developed by Ridom GmbH (Münster, Germany).

### 2.4. Susceptibility Testing

All strains were tested using disk-diffusion antimicrobial susceptibility methods on Mueller-Hinton agar plates, according to the current guidelines of the European Committee on Antimicrobial Susceptibility Testing (Clinical breakpoints tables v. 8.1; http://www.eucast.org v.8.1, accessed on 13 June 2018) A strain was considered drug-resistant if it was non-susceptible to one or more agent in any antimicrobial categories.

### 2.5. SCCmec Typing

Staphylococcal cassette chromosome mec (SCC*mec*) typing was performed as described by Kondo et al. (10). The following *S. aureus* strains were used as controls: ATCC BAA1762 (SCC*mec IV*), ATCC BAA2094 (SCC*mec V*) and ATCC BAA1681 (SCC*mecII*).

### 2.6. Pulsed-Field Gel Electrophoresis (PFGE)

The analysis of a genetic similarity between the *S. aureus* isolates was performed using PFGE in accordance with a protocol published by McDougal et al. [23] Restriction enzyme digestion was performed with 25 U of *SmaI* enzyme in Tango buffer (ThermoScientific, Waltham, MA, USA). Electrophoresis was conducted in a CHEFIII PFGE unit applying the parameters as in Supplementary Materials File S1. Isolates with more than 95% of similarity were clustered together as identical.

### 2.7. Statistical Methods

We have constructed a binary logistic regression model of the drug-resistance of the isolated *Staphylococcus aureus* strains, with 1 meaning that a strain was drug-resistant, and 0—that it was drug-sensitive. The independent variables were the age group of a patient and the need (taking the value of 1) or no need (0) of hospitalisation. A one-way ANOVA, with post-hoc tests, the Bonferroni correction and a classification tree (with a 50/50 a priori probability assumed), has shown statistically significant differences between the age groups. The statistical analysis was performed by means of the IBM SPSS Version 24 (http://www-01.ibm.com/software/uk/analytics/spss/, RRID:SCR_002865, accessed on 26 March 2014). The Odds ratios (Ors) and the 95% confidence intervals (Cis) were calculated at: https://www.medcalc.org/calc/odds_ratio.php (accessed on 28 March 2014).

### 2.8. Ethics

This work has been approved by the Bioethics Committee of Jagiellonian University Medical College in Cracow, Poland (approval no KBET/227/B/2012). All data analysed in the course of this study had previously been anonymised.

## 3. Results

**Figure 1:**
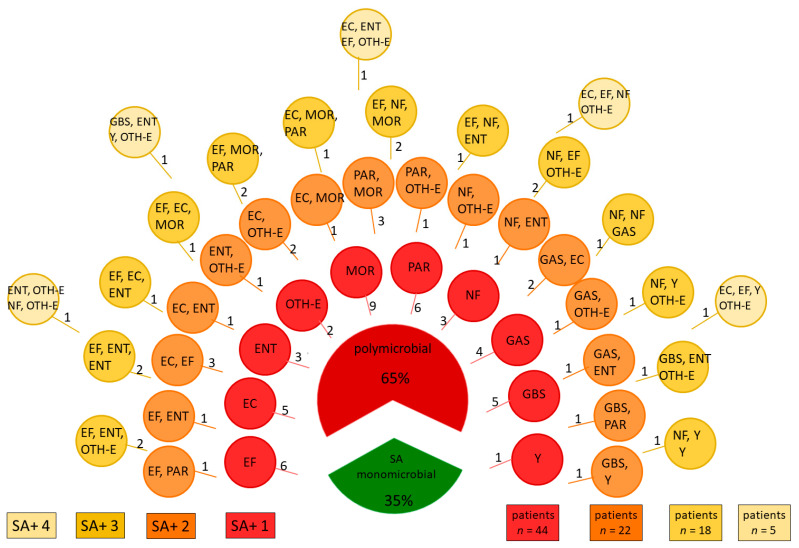
Microbiota isolated from infected venous ulcers from 143 patients, numbers indicate numbers of patients. Legend*:* EC *Escherichia coli*; EF *Enterococcus faecalis*; ENT *Enterobacter* spp.; GAS *Streptococcus group A*; GBS *Streptococcus group B*; MOR bacteria from *Morganellaceae* family; NF other non fermenting bacteria; OTH-E other bacteria from *Enterobacteriaceae* family*;* PAR *Pseudomonas aeruginosa*; SA *Staphylococcus aureus*; Y yeast. *n*—number of patients in the group with one, two, three or four pathogens in addition to SA in the polymicrobial group, respectively.

The median age of the studied population was 62 (interquartile range, IQR 56;77), 27.3% were aged over 75, 53.2% were women. In the microbiological diagnostics of the 143 patient samples, we identified 50 cases where *Staphylococcus aureus* was the single aetiological factor (34.9%), 45 cases (31.5%) in which it was accompanied by another factor, and 48 (33.6%) cases with three or more aetiological factors (Figure 1). Apart from *S. aureus*, we identified other pathogens as in Table 1.

In four samples, we identified the presence of 5 microbe species which we could not identify, and those were excluded from the analysis.

MRSA screening and eradication based on strict rules had not been routinely performed (before surgical procedures or at the admission to the hospital) in any of the analysed hospitals.

Among the virulence factors, the pvl gene was observed the least frequently, in only 1.4% strains. The majority of isolates (68.5%) possessed the lukE gene, with no significant difference in prevalence between sensitive and drug-resistant strains. No difference was also detected in the prevalence of the tsst-1 (11.9%) and etA/B (16.0%) genes (*p*-value < 0.05) (Table 2). In the group of patients over 80 years of age, the ratio of resistance to susceptible strains increases significantly compared to the 60–75 age group. There was no association between the occurrence of drug-resistance strains and the gender of the patients. The drug-resistance was significantly more common in the hospitalised patients, but the type of care they received (medical or surgical) did not matter (Table 2).

Strains were generally susceptible to trimethoprim/sulfamethoxazole (94.6%) and fully susceptible to vancomycin, tigecycline and linezolid. The MIC50 for vancomycin was equal 1.0 mg/L and MIC90 was 1.5 mg/L. The MIC50 for tigecycline was 0.125 mg/mL and the MIC90 was 0.19 mg/mL. The resistance to other antibiotics was moderate or low, and the highest resistance was found for tobramycin (27.3%) and tetracycline (24.5%) (Table 3). Hospitalisation augmented the multidrug-resistance prevalence in comparison to the ambulatory patients but without statistical significance. Specifically, the resistance to chloramphenicol was two times higher in the hospitalised patients: 13.7% vs. 6.5%. The resistance to quinupristin–dalfopristin among the studied isolates has been observed in the hospitalised patients only, and amounted to 5.3% (Table 3).

A constitutive macrolide-lincosamide-streptogramin B (MLSB) resistance mechanism was detected among 28 (19.6%) isolates, mostly among non-hospitalized patients: OR 9.1; 95%CI 1.17–71.02. MRSA was detected in 11.9% of isolates, in both ambulatory and hospital care (Table 2).

The significant features (*p* < 0.001) of the logistic regression model of the presence of a drug-resistant *S. aureus* was age group.

**Figure 2:**
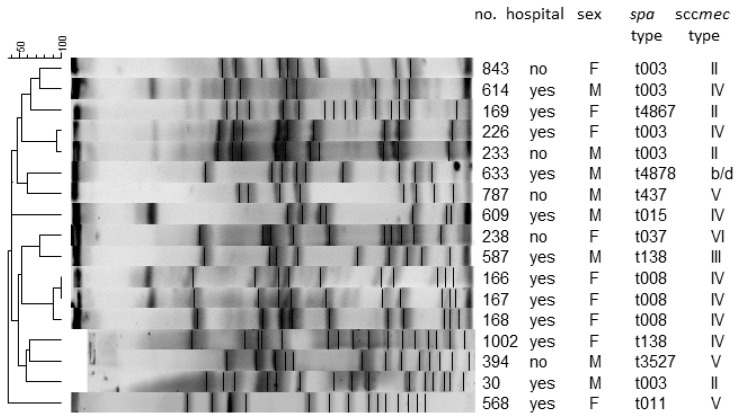
Analysis of the genetic similarity between S. aureus isolates by the PFGE (Pulsed-Field Gel Electrophoresis) method based on spa and sccmec genes.

*S. aureus* isolates showed very different pulsotypes, no dominant clones were detected. Cluster analysis based on PFGE showed that pulsotypes were similar in less than 70%, suggesting a genotypically varied population (data not shown). Among MRSA strains, three isolates with identical pulsotypes were found. They came from different patients of the same LTCF unit and had the same *spa*-type (t008) and SCC*mec* type (IV) (Figure 2).

Overall, 10 different *spa* types (mostly t003, 29.4%) and five SCC*mec* (mostly SCC*mec* IV, 41.2%) were observed (Figure 2) in all MRSA strains.

## 4. Discussion

*S. aureus* is the most important aetiological agent in wound infections, including chronic wounds [24,25]. However, when a chronic wound is infected, bacteria often form polymicrobial biofilms, unlike in the case of acute wounds, such as surgical site infections. In two thirds of the studied population, no more than two microorganisms were isolated. Apart from *S. aureus*, the bacteria from *Morganellaceae family* were most frequently detected. In the remaining one third of cases, polymicrobial biofilms with a higher virulence compared to single-species biofilms were observed [26]. The common pathogens were *Pseudomonas aeruginosa*, Gram-positive cocci (GAS, GBS, EC, EF) and yeast-like fungi.

Microorganisms in chronic wounds prolong the healing [27]. Besides direct damage they cause to the tissue, bacteria attract leukocytes and activate inflammatory cytokines, proteases, and associated reactive oxygen species, thus both initiating and maintaining inflammatory cascades [28,29].

The strains we have analysed rarely contained genes responsible for specific types of virulence, which may point to their potentially low pathogenicity. The observed virulence was much lower than in previously studied Polish strains, isolated from invasive infections, such as surgical site infections. [18] The results do not show any correlation between the virulence and the presence of certain genes, in particular, between pvl genes and the *spa* type. The drug-resistance of the studied *S. aureus* strains does not raise any particular concerns either. Only one in five strains was multidrug-resistant, mostly to erythromycin and clindamycin (MLSB), as well as to tetracyclines and aminoglycosides. 1/3 of the strains were resistant to two or three antibiotic groups, but their resistance was weaker than in other patient groups in southern Poland, such as very-low-birth-weight infants or the geriatric patients of ambulatory care [18,30]. The high level of MLSB may result from a particularly very high consumption of macrolides, almost the highest in Poland compared to other EU at more than five DDD per 1000 inhabitants per day, while in the EU not more than three [17].

The author observed the high prevalence of *S. aureus* resistance to aminoglycosides and tetracyclines. These resistances are in part related to SCC*mec* modifications, but also other resistance mechanisms are known [31]. A lack of resistance to the group of antibiotics was observed significantly more often in the group of people not remaining for hospitalization. This suggests good practice towards rapid patient discharge due to the risk of infection or co-infection with potentially resistant pathogens [32]. This is important in preventing the spread of resistant infections, which was partly confirmed by our study, due to the coexistence of the same *spa*-type and SCC*mec* type in three patients. All staphylococcal strains were susceptible to vancomycin and linezolid. This is a positive result in the presented data due to the aforementioned abuse of antimicrobial agents in the Polish population, the more so as these drugs are more and more often the last alternative in the treatment of highly resistant strains. High resistance to fluoroquinolones in the obtained results (ciprofloxacin 17.5% and moxifloxacine 12.6%) may result from the high consumption of this group of antibiotics. In Europe, quinolones are the third most frequently used group of antibiotics after penicillins and beta-lactams [33]. The argument for the influence of exposure to antimicrobial agents is supported by an increase in the ratio of resistant to susceptible pathogens in the oldest group of patients in our study.

Effective surveillance of *S. aureus* can be achieved through a combination of the traditional or cultural standard methods and several molecular techniques, manly polymerase chain reactions (PCR)-based. Molecular techniques have been widely used in molecular epidemiology or outbreak investigation, and have the advantage that, they are rapid, less laborious, and more sensitive, specific and efficient compared to the conventional method. Typing of microorganisms covers the methods which enable to reproduce the transmission routes of pathogens as well as compare them with global spreading of specially virulent strains and the most important in everyday practice is use the multi locus seqence typing (MLST)—a reference method for establishing the basic genetic structure of *S. aureus* population, which is dominated by several large clone complexes and includes several hundred sequence types (ST)—or *spa* typing, based on the sequencing of short repetitive sequences of the polymorphic X region from the gene encoding protein A [34,35]. The ‘gold standard’ for the typing of *Staphylococcus aureus* is pulsed-field gel electrophoresis (PFGE) which allow to determine the spread of mi-croorganisms, which relies on separating the DNA fragments after restriction cutting, very often used for outbreak investigation in drug-resistant epidemic pathogens [36].

The genetic typing of MRSA and PFGE pointed to a high diversity among the strains, which means that colonisation was mostly not associated with the hospital environment. An MRSA clone was only found in three patients of one LTCF. The *spa* typing has confirmed the *spa* type *t003* to be the most predominant among MRSA strains at hospitals in Southern Poland, as reported previously [37].

One of the problems we had to face when designing this study was how to collect the materials for analysis. The microbiological diagnostics of chronic wound infections largely depend on the way in which materials were collected. What is characteristic about chronic wounds is the presence of diverse bacteria on the surface, observed as a biofilm. The microbes in the biofilm are adapted to difficult conditions, and can survive different kinds of treatment, including the use of antibiotics [38,39]. A microbiological analysis should provide credible information on the aetiological factors of an infection, which are often located deep under the surface. That is why using the invasive methods, such as tissue aspirates or biopsies, rather than wound swabs is advised [40,41,42]. Invasive methods are optimal for a quantitative microbiology analysis and facilitate the identification of the aetiological factors of infections, no matter where they are located. However, they also have some downsides, since they are painful for patients and they disturb the wound healing. They may also cause the contamination of deeper located tissues or trigger a haemorrhage. Moreover, the specimen collection has to be performed by an experienced doctor, and an anaesthetic needs to be applied. That is why swabs are used so frequently, and why we decided to resort to them as well. They are non-invasive and can be collected by a nurse, not necessarily a doctor, in any conditions [43].

All in all, it seems that the best solution would be to use a method that is most appropriate given the specific conditions of each patient. The invasive (biopsies) and non-invasive (swabs) methods could complement one another in the clinical diagnostics. A wound swab may, in some situations, actually yield more meaningful results. Doctors should, then, always take advice from a microbiology consultant on the best method for specimen collection once they recognise the clinical signs of an infection [44].

One of the weaknesses of our study was the lack of confirmed presence of anaerobes and fastidious bacteria in the analysed materials [45,46]. We must also point out that the drug sensitivity was tested in vitro, with no regard to the specific conditions of a biofilm.

## 5. Conclusions

First of all, in our study, we pay attention to the identification of the same strains only among hospital patients. Therefore, special attention should be paid to the prevention of pathogen transmission between patients using the disinfection algorithms recommended by WHO.

The risk of multi-drug-resistant strains increases with age and is significantly the highest in the group of the oldest people. Mention was made of a possible coincidence of this phenomenon with increasing consumption of antibiotics. Therefore, special attention should be paid to rational antibiotic therapy, taking into account the guidelines for the use of antibiotic therapy in chronic wounds.

The optimal method of collecting material for research is a biopsy, but a properly performed swab can also provide clinically significant data.

Due to the constantly growing resistance of pathogens to the antimicrobial agents used, it can be concluded that this phenomenon will now be even more intense than in the results obtained.

## Figures and Tables

**Table 1:** Other isolated pathogens.

Identified Pathogen	*n*
*Enterococcus faecalis* (EF)	30
*Streptococcus agalactiae* (GBS)	10
*Streptococcus pyogenes* (GAS)	9
*Escherichia coli* (EC)	19
*Enterobacter* spp (ENT)	19
**OTH-E: Other Bacteria from *Enterobacteriaceae Family***	
*Klebsiella* spp.	12
*Serratia* spp.	3
*Citrobacter* spp.	6
***Morganellaceae Family* (MOR)**	
*Proteus* spp.	17
*Morganella morgani*	3
*Providentia regretti*	1
*Pseudomonas aeruginosa* (PAR)	15
**Other Non-Fermenting Bacteria (NF)**	
*Acinetobacter* spp.	13
*Stenotrophomonas maltophilia*	4
*Candida* spp. (Y)	7

**Table 2:** Characteristics of the study group according to drug sensitivity of *Staphylococcus aureus.*

Characteristics of the Study Group	Total *n* = 143	Sensitivity to Antibiotics		OR (95% CI)
		Yes *n* = 70	No *n* = 73	
**Age (Years) by Categories *n* [%]**				
≤59 years	45 (31.5)	23 (33.3)	22 (30.1)	0.8 (0.36–1.73)
60–75 years	58 (40.6)	33 (47.8)	25 (34.2)	ref.
76–80 years	16 (11.2)	6 (8.7)	10 (13.7)	0.5 (0.15–1.41)
≥81 years	24 (16.8)	7 (10.1)	16 (21.9)	0.3 (0.12–0.93)
**Gender *n* [%]**				
Female	76 (53.2)	34 (48.6)	42 (57.5)	0.7 (0.36–1.35)
Male (ref.)	67 (46.9)	36 (51.4)	31 (42.5)	
**Ambulatory Care *n* [%]**				
yes	48 (32.2)	29 (41.4)	19 (26.0)	2.0 (0.99–4.08)
no	95 (66.4)	41 (58.6)	54 (74.0)	
**Hospital Stay *n* [%]**				
surgical wards	61 (42.7)	26 (37.1)	35 (47.8)	0.9 (0.40–2.19)
non-surgical wards or LTCF	34 (23.8)	15 (21.4)	19 (13.3)	
**The Presence of Resistance Genes *n* [;%], YES**				
*mecA*	17 (11.9)	n/a	17 (11.9)	n/a
*ermA*	8 (5.6)	n/a	8 (5.6)	n/a
*ermB*	2 (1.4)	n/a	2 (1.4)	n/a
*msr*	1 (0.7)	n/a	1 (0.7)	n/a
**The Presence of Various Virulence Factors Genes *n* [;%], YES**				
*lukE*	98 (68.5)	45 (64.3)	53 (72.6)	0.7 (0.33–1.38)
*tsst-1*	17 (11.9)	9 (12.9)	8 (11.0)	1.2 (0.43–3.30)
*pvl*	2 (1.4)	1 (1.4)	1 (1.4)	1.0 (0.06–17.01)
*etA/B*	23 (16.1)	10 (14.3)	13 (17.8)	0.8 (0.31–1.89)

Legend: etA/B exfoliative toxin A and/or B; LTCF long term care facilities; lukE LukDE leukocidin; n/a not applicable; OR—Odds ratio; (95%CI) 95% Confidence Interval; pvl Panton-Valentine leukocidin; ref.—reference; tsst-1 toxic shock syndrome toxin-1.

**Table 3:** Drug-resistance of *Staphylococcus aureus* isolated from hospitalised and non-hospitalised patients.

Antimicrobial Category	Antimicrobial Agent	Total *n* = 143	Hospital Stay		OR (95%CI)
			Yes, *n* = 95	No, *n* = 48	
Aminoglycosides	Gentamicin	24 (16.8)	16 (16.8)	8 (16.7)	0.9 (0.38–2.45)
	Amikacin	29 (20.2)	19 (20.0)	10 (20.8)	2.1 (0.71–5.89)
	Tobramycin	39 (27.3)	27 (28.4)	12 (25.0)	1.1 (0.51–2.49)
Anti-MRSA cephalosporins	Ceftaroline	0 (0)	0 (0.0)	0 (0.0)	n/a
Fluoroquinolones	Ciprofloxacin	25 (17.5)	20 (21.1)	5 (10.4)	2.2 (0.76–6.26)
	Moxifloxacin	18 (12.6)	14 (14.7)	4 (8.3)	0.5 (0.23–1.33)
Folate pathway inhibitors	Trimethoprim/Sulfamethoxazole	8 (5.6)	6 (6/3)	2 (4.2)	1.5 (0.29–7.65)
Lincosamides	Clindamycin	29 (20.2)	18 (18.9)	11 (22.9)	0.7 (0.32–1.74)
Macrolides	Erythromycin	31 (21.6)	17 (17.9)	14 (29.2)	0.6 (0.27–1.43)
Glycopeptides	Vancomycin	0 (0)	0 (0.0)	0 (0.0)	n/a
Glycylcyclines	Tigecycline	0 (0)	0 (0.0)	0 (0.0)	n/a
Oxazolidinones	Linezolid	0 (0)	0 (0.0)	0 (0.0)	n/a
Phenicols	Chloramphenicol	16 (11.2)	13 (13.7)	3 (6.2)	0.3 (0.13–0.69)
Streptogramins	Quinupristin-dalfopristin	0 (0)	0 (0.0)	0 (0.0)	n/a
Tetracyclines	Tetracycline	35 (24.5)	27 (28.4)	8 (16.7)	1.9 (0.78–4.56)
	Doxycycline	19 (13.3)	13 (13.7)	6 (12.5)	1.1 (0.37–2.99)
Others (O)	Mupirocin	8 (5.6)	5 (5.3)	3 (6.2)	0.8 (0.18–3.49)
**Multidrug Resistance *n* (;%)**					
MRSA, yes		17 (11.9)	12 (12.6)	5 (10.4)	1.2 (0.39–3.59)
MLSB, yes		28 (19.6)	16 (16.8)	12 (25.0)	9.1 (1.17–71.02)
**Non-Susceptible to Antimicrobial Categories *n* (;%)**					
0 categories (fully susceptible)		70 (49)	41 (43.2)	29 (60.4)	0.4 (0.22–0.92)
1 category		26 (18.2)	23 (24.2)	3 (6.2)	4.8 (1.38–17.06)
Aminoglycosides		9 (6.3)	7 (7.4)	2 (4.2)	
Tetracyclines		9 (6.3)	9 (9.5)	0 (0.0)	
Phenicols		4 (2.8)	4 (4.2)	0 (0.0)	
Fluoroquinolones		2 (1.4)	2 (2.1)	0 (0.0)	
Lincosamides		1 (0.7)	1 (1.1)	0 (0.0)	
Macrolides		1 (0.7)	0 (0.0)	1 (2.1)	
2 categories		19 (13.3)	11 (11.6)	8 (16.7)	0.6 (0.23–1.67)
Macrolides + Lincosamides		5 (3.5)	2 (2.1)	3 (6.3)	
Tetracyclines + Phenicols		3 (2.1)	2 (2.1)	1 (2.1)	
Aminoglycosides + Fluoroquinolones		3 (2.1)	3 (3.2)	0 (0.0)	
Tetracyclines + Fluoroquinolones		2 (1.4)	2 (2.1)	0 (0.0)	
Aminoglycosides + Tetracycline		2 (1.4)	1 (1.1)	1 (2.1)	
Aminoglycosides A+ Phenicols		2 (1.4)	1 (1.1)	1 (2.1)	
Tetracyclines + Macrolides		1 (0.7)	0 (0.0)	1 (2.1)	
Aminoglycosides + Macrolides		1 (0.7)	0 (0.0)	1 (2.1)	
3 categories or more		28 (19.6)	19 (20.0)	8 (16.7)	1.2 (0.48–2.96)

OR (95%CI) 95% confidence intervals of odds ratio; MRSA—methicillin resistant *Staphylococcus aureus*; MLSB—Macrolide-lincosamide-streptogramin B resistance phenotype.

## Data Availability

The datasets generated during and/or analysed during the current study are available from the corresponding author on reasonable request.

## Supplementary Materials

The following are available online at https://www.mdpi.com/article/10.3390/ijerph18094662/s1, File S1: precise Methodology description.
