# Supplementary material for: Virulence and Drug-Resistance of Staphylococcus aureus Strains Isolated from Venous Ulcers in Polish Patients
Source: Int J Environ Res Public Health. 2021 Apr 27;18(9):4662. doi: 10.3390/ijerph18094662 (PMC8124697; doi:10.3390/ijerph18094662)
Supplement: Supplementary file 1 [file ijerph-18-04662-s001.zip › ijerph-1141293-supplementary.pdf]

Supplementary material

Susceptibility testing

All strains were tested using disk-diffusion antimicrobial susceptibility methods on Mueller-Hinton agar plates, according to the current guidelines of the European Committee on Antimicrobial Susceptibility Testing (Clinical breakpoints tables v. 8.1; http://www.eucast.org v.8.1). Antibiotics used in this study included gentamycin (10 µg), tobramycin (10 µg), amikacin (30 µg). ceftaroline (5 µg), ciprofloxacin (5 µg), moxifloxacin (5 µg), trimethoprim/sulfamethoxazole (1.25-23.75 µg), erythromycin (15 µg), clindamycin (2 µg), linezolid (10 µg), chloramphenicol (30 µg), tetracycline (30 µg), mupirocin (200 µg). All disks were obtained from Oxoid (Basingstoke, United Kingdom). E-tests for doxycycline (0.016 do 256), quinupristin-dalfopristin (0.002-32 µg) (Liofilchem, Italy) and tigecycline (0.016-256 µg) (bioMerieux, France) were performed on all the isolates, while the e-test for vancomycin (0.016-256 µg) (bioMerieux, France) was performed only for MRSA isolates.

A strain was considered drug-resistant if it was non-susceptible to one or more agent in any antimicrobial categories.

MRSA and MLS_B phenotype

The MRSA resistance phenotype was detected using a cefoxitin disc (30 µg), according to the current guidelines of the European Committee on Antimicrobial Susceptibility Testing (EUCAST, clinical breakpoint tables v. 6.0; http://www.eucast.org/clinical_breakpoints/, accessed 01.01.2016). The macrolide-lincosamide-streptogramin B (MLS_B) resistance phenotype of the isolates was determined according to a previously published protocol by Leclercq (1).

DNA isolation

DNA was extracted from the isolates using the Genomic Mini kit (A&A Biotechnology, Gdynia, Poland), according to the manufacturer's instructions.

Polymerase chain reaction (PCR) screening for resistance genes

MRSA phenotype was determined by the detection of the *mec*A gene in PCR amplification using previously published primers (2). Genes involved in erythromycin resistance (*erm*A, *erm*B, *erm*C and *msr*) were detected by multiplex PCR and amplification of a 456 bp fragment of the *mup*A gene performed by single PCR (3,4). Relevant positive (S. aureus ATCC 33591, ATCC BAA-1708) were included. Water was used for the negative control.

Virulence factor screening

S. aureus isolates were checked for the presence of selected virulence genes: *tsst* (toxic shock syndrome toxin), *sea, seb, sec, seg, seh, sei, sej* (staphylococcal enterotoxins A, B, C, G, H, I, J), *eta, etb* (exfoliative toxins A i B), *luk*E (LukDE leukocidin), *hla* (α-hemolysin) and *pvl* (Panton-Valentine leucocidin) using PCR with previously published primers (5–7). The results were considered positive if the amplification product was of the expected molecular size (*tsst* – 559 bp, *sea* – 520bp, *seb* - 667bp, *sec* - 284bp, *seg* - 328bp, *seh* - 359bp, *sei* – 466bp, *sej*-142bp, *eta*- 119bp, *etb* – 200bp, *luk*E- 269bp, *pvl*- 433bp, *hla* – 274bp). The following *S.aureus* strains were used as positive controls: 2535/07 (*eta*+, *etb*+, *seg*+, *sei*+), 8977/99 (*sea*+, *sec*+, *seg*+, *sei*+), 6616/09 (*seb*+, *tsst*+, *pvl*+), 2027/06 (*sea*+, *seh*+), 1034/05 (*sea*+, *seg*+, *sei*+, *sej*+). The strains used as controls were kindly provided by Prof. Marek Gniadkowski, National Medicines Institute, Warsaw, Poland.

Pulsed-field gel electrophoresis (PFGE)

The analysis of a genetic similarity between the *S. aureus* isolates was performed using PFGE in accordance with a protocol published by McDougal et al. (8). Restriction enzyme digestion was performed with 25 U of *Sma*I enzyme in Tango buffer (ThermoScientific, USA). Electrophoresis was conducted in a CHEFIII PFGE unit applying the parameters: block1 starting pulse 5 s, ending pulse 12 s, voltage 6V/cm, running time 11h, block 2 starting pulse 20 s, ending pulse 60 s, voltage 6V/cm, running time 13h. The Gel Compar (AppliedMaths, Sint-Martens-Latem, Belgium) was used for cluster analysis, using the Dice coefficient and unweighted pair group method with arithmetic mean. Isolates with more than 95% of similarity were clustered together as identical.

***Spa* typing**

To determine the *spa* type of the polymorphic X-region of the *S. aureus* protein A, the *spa* gene was amplified by PCR using a previously published method (9). The amplicons were analysed by electrophoresis on a 1.5% agarose gel. The sequencing of PCR products was subcontracted to an external laboratory (Genomed, Warsaw, Poland). The nucleotide sequences were analysed to assign the isolates to various types using the *spa* typing website Ridom SpaServer (http://www.spaserver.ridom.de), developed by Ridom GmbH (Münster, Germany).

SCC*mec* typing

Staphylococcal cassette chromosome mec (SCCmec) typing was performed as described by Kondo et al. (10). The following S. aureus strains were used as controls: ATCC BAA1762 (SCC*mec* IV), ATCC BAA2094 (SCC*mec* V) and ATCC BAA1681 (SCC*mec*II).

References:

1. Leclercq R. Mechanisms of Resistance to Macrolides and Lincosamides: Nature of the

Resistance Elements and Their Clinical Implications. Clin Infect Dis. 2002;34(4):482–92.

2. Pereira EM, Schuenck RP, Malvar KL, Iorio NLP, Matos PDM, Olendzki AN, et al. Staphylococcus aureus, Staphylococcus epidermidis and Staphylococcus haemolyticus: Methicillin-resistant isolates are detected directly in blood cultures by multiplex PCR. Microbiol Res. 2010;165(3):243–9.

3. Sutcliffe J, Grebe T, Tait-Kamradt A, Wondrack L. Detection of erythromycin-resistant determinants by PCR. Antimicrob Agents Chemother. 1996;40(11):2562–6.

4. Anthony RM, Connor AM, Power EGM, French GL. Use of the polymerase chain reaction for rapid detection of high-level mupirocin resistance in staphylococci. Eur J Clin Microbiol Infect Dis. 1999;18(1):30–4.

5. Johnson WM, Tyler SD, Ewan EP, Ashton FE, Pollard DR, Rozee KR. Detection of genes for enterotoxins, exfoliative toxins, and toxic shock syndrome toxin 1 in Staphylococcus aureus by the polymerase chain reaction. J Clin Microbiol. 1991;29(3):426–30.

6. Lina G, Piemont Y, Godail-Gamot F, Bes M, Peter M-O, Gauduchon V, et al. Involvement of Panton-Valentine Leukocidin--Producing Staphylococcus aureus in Primary Skin Infections and Pneumonia. Clin Infect Dis. 1999;29(5):1128–32.

7. Løvseth A, Loncarevic S, Berdal KG. Modified multiplex PCR method for detection of pyrogenic exotoxin genes in staphylococcal isolates. J Clin Microbiol. 2004 Aug 1;42(8):3869–72.

8. Mcdougal LK, Mcdougal LK, Steward CD, Steward CD, Killgore GE, Killgore GE, et al. Pulsed-Field Gel Electrophoresis Typing of Oxacillin-Resistant Staphylococcus aureus Isolates from the United States: Establishing a National Database. J Clin Microbiol. 2003;41(11):5113–20.

9. Harmsen D, Claus H, Witte W, Claus H, Turnwald D, Vogel U. Typing of Methicillin-Resistant. Society. 2003;41(12):5442–8.

10. Kondo Y, Ito T, Ma XX, Watanabe S, Kreiswirth BN, Etienne J, et al. Combination of multiplex PCRs for staphylococcal cassette chromosome mec type assignment: Rapid identification system for mec, ccr, and major differences in junkyard regions. Antimicrob Agents Chemother. 2007;51(1):264–74.
